# *COL1A2* polymorphic markers confer an increased risk of neovascular age-related macular degeneration in a Han Chinese population

**Published:** 2012-06-30

**Authors:** Chengguo Zuo, Feng Wen, Meng Li, Xiongze Zhang, Hui Chen, Kunfang Wu, Renpan Zeng

**Affiliations:** State Key Laboratory of Ophthalmology, Zhongshan Ophthalmic Center, Sun Yat-sen University, Guangzhou, China

## Abstract

**Purpose:**

We have previously documented that neovascular age-related macular degeneration (nAMD) and polypoidal choroidal vasculopathy (PCV) have multiple different clinical and genetic characteristics. In this study, we investigated the association of rs42524 in the alpha-2 type I collagen (*COL1A2*) gene, which has been identified as a risk variant for intracranial aneurysm, with nAMD and PCV in a Han Chinese population.

**Methods:**

The study prospectively recruited 195 patients with PCV, 136 patients with nAMD, and 181 control individuals. We genotyped the rs42524 polymorphism of *COL1A2* using the Multiplex SNaPshot System and direct DNA sequencing. Genotype and allele frequencies were evaluated with PLINK software.

**Results:**

The rs42524 polymorphism was modestly significantly associated with nAMD [minor allele: G, p(allelic)=0.04253, odds ratio=0.5285 (95% confidence interval: 0.2832–0.9866)], but not with PCV [minor allele: G, p(allelic)=0.4164, odds ratio=1.2110 (95% confidence interval: 0.7631–1.9210)]. The p**values for the additive model were significant for nAMD but not for the dominant or recessive models. None of the models for PCV were statistically significant. The size of our sample cohort resulted in a post hoc power of more than 80% to detect associations of rs42524 with nAMD and PCV.

**Conclusions:**

The rs42524 polymorphism is a risk allele for nAMD in a Han Chinese population. rs42524 in *COL1A2* confers different levels of susceptibility to nAMD and PCV.

## Introduction

Polypoidal choroidal vasculopathy (PCV) is characterized by polyp-like terminal aneurysmal dilations with or without branching choroidal vessels [1-4]. Although the visual prognoses and potential responses to treatment differ between PCV and neovascular age-related macular degeneration (nAMD), they share several common characteristics, including subretinal hemorrhage, pigment epithelial detachment (PED), and increased prevalence in people more than 50 years of age [1,2,5]. In view of the similarities between nAMD and PCV, several studies have investigated the relationship between the genetic variants associated with both conditions. Although some studies have found a shared genetic background [6-10], others have found little to no genetic similarity [11,12].

Although the genetics of nAMD have been well studied, investigations into the genes encoding the structural proteins involved in this disease are limited [13-16]. However, histopathological studies of the choroidal neovascular (CNV) membranes of AMD have found abnormal vessels surrounded by fibrin-like materials [17]. In contrast, the genetic investigation of PCV is just beginning, and only a few studies have conducted single nucleotide polymorphism (SNP) analyses of PCV [9,12,18-21]. Kuroiwa et al. observed the histopathologic changes of PCV and found vessel wall sclerosis and an increase in basement membrane-like materials and collagen fibers within the wall of polypoidal lesions [22]. Nakashizuka et al. [23] and Okubo et al. [24] also investigated the histopathologic characteristics of PCV lesions and found vessel wall destruction that manifested as wall thickening and apparent hyaline degeneration. These pathologic findings provide an important clue to the possible relationship between nAMD, PCV, and vessel wall destruction.

Collagen destruction can result in a decrease in vessel integrity and an increase in vessel permeability [25]. Type I collagen is the critical component required for maintaining vessel wall elasticity and is an important component of the extracellular matrix [26]. Collagen fiber disintegration in pericellular connective tissue decreases the accumulation of connective tissue in vessel walls, which in turn decreases wall flexibility. This decrease in wall flexibility has been associated with CNV, PCV, and intracranial aneurysm (IA).

Type I collagen is the most abundant connective tissue protein in human organ systems. Type I collagen consists of two alpha-1 and one alpha-2 chains [27]. The alpha-2 type I collagen (*COL1A2*) gene is located in the 7q22.1 locus and encodes the pro-alpha 2 chain protein. The rs42524 polymorphism in *COL1A2* results in an amino acid substitution, Ala to Pro, at amino acid position 459 and therefore influences the integrity of type I collagen, decreases vessel wall rigidity, and eventually causes the destruction of blood vessel walls [28].

To our knowledge, this is the first investigation into the association between *COL1A2*, PCV, and nAMD. The purpose of our study was to assess the associations of rs42524 with PCV and nAMD in a Han Chinese population.

## Methods

### Study population

A prospective study was conducted on 512 participants including 195 patients with PCV, 136 patients with nAMD, and 181 control individuals. Each participant was examined at the Zhongshan Ophthalmic Center (Guangzhou, China). The patients’ medical histories were reviewed. All patients underwent visual acuity testing, slit-lamp biomicroscopy, and ophthalmoscopic examination. Color fundus photography, fluorescein angiography, and indocyanine green angiography were performed in both eyes of the patients with PCV and nAMD. The diagnostic criteria for PCV were polypoidal choroidal vascular dilations with or without branching inner choroidal vessels on indocyanine green angiography [29]. Patients with other neovascularized maculopathies such as retinal angiomatous proliferation, pathological myopia, angioid streaks, central serous chorioretinopathy, presumed ocular histoplasmosis, and other retinal or choroidal diseases that could account for CNV were excluded. All control subjects were unrelated to the case subjects and were aged ≥50 years. All subjects with macular changes such as drusen or pigment abnormalities, macular degeneration of any cause, or media opacities preventing clear observation of the fundus were excluded from recruitment.

The study protocol was approved by the institutional review board at the Zhongshan Ophthalmic Center of Sun Yat-sen University. Informed consent was obtained from all patients before angiography. All procedures adhered to the tenets of the Declaration of Helsinki.

### Single nucleotide polymorphism genotyping

Genomic DNA from peripheral blood samples was isolated using the NucleoSpin Blood XL kit (Macherey-Nagel GmbH & Co., KG Düren, Germany) and stored at −20 °C. Genotyping was performed using a PCR restriction fragment length polymorphism assay. Direct sequencing was performed to confirm the restriction patterns for 10% of the samples. rs42524 in *COL1A2* was genotyped with the Multiplex SNaPshot System with an ABI 3730XL Genetic Analyzer (Applied Biosystems, Foster City, CA). SNP genotypes were determined with GeneMapper software V4.1 (Applied Biosystems). The primer sequences used for the SNP were as follows: forward 5′-CAA GGT GGA AAA GGT GAA CAG-3′ and reverse 5′-AGC TCA ATA GGC TGA CCA AAG-3′. The extension primer was 5′-TTT TTT TTT TTT TTT TTT TTT TTT GGA AGC CTG GAG GAC CAG-3′.

### Statistics

A statistical analysis of the data was performed using Statistical Package for the Social Sciences (SPSS) software (version 16.0, SPSS Inc., Chicago, IL). Baseline characteristics between the cases and controls were compared using unpaired Student *t* tests for means and chi-square tests for proportions. An exact test implemented in the PLINK v1.07 software package was used to test for deviations from the Hardy-Weinberg equilibrium [30]. The minor allele was determined based on all case and control subjects. Allele frequencies were compared between cases and controls using chi-square tests along with PLINK as previously described [14]. The logistic option in PLINK was used to provide a test based on logistic regression for the genotypic additive model, and the model option in PLINK was used to provide a chi-square test for the dominant and recessive models. The odds ratio and corresponding 95% confidence interval (CI) were calculated relative to the minor allele and the wild-type homozygote. A p-value <0.05 was considered statistically significant. The G* power 3 program (Erdfelder, Faul, & Buchner, Mannheim, Germany) [31] was used to perform post-hoc power analyses.

## Results

A total of 512 subjects participated in this study, including 136 patients with nAMD, 195 patients with PCV, and 181 control individuals. The percentage of male patients in the nAMD, PCV, and control populations was 63.2% (86 cases), 66.7% (130 cases), and 61.9% (112 cases), respectively. There was no significant difference between the control group and the PCV (p=0.333) or nAMD (p=0.805) group regarding gender. The mean age of the PCV group (64±8.75 years) was significantly lower than that of the control group (68±9.18 years; p<0.001). There was no significant age difference between the nAMD group (67±9.29 years) and the control group.

Genotypes were determined using a PCR restriction fragment length polymorphism assay in all patients and were confirmed with direct sequencing in a subset. The population tested in this study did not show any significant deviation from the Hardy–Weinberg equilibrium for the observed genotype (p>0.1000; Table 1).

**Table 1:** Association test for the minor allele frequency of the rs42524 polymorphism in nAMD, PCV and control subjects

**Status**	Minor allele*	**HWE**	**MAF**	**OR (95%CI)**	**p-value**
nAMD		1	0.0804	0.5285 (0.2832–0.9866)	0.0425
PCV		0.7345	0.109	1.2110 (0.7631–1.9210)	0.4164
Control	G	0.6879	0.0947		

nAMD=neovascular age-related macular degeneration; PCV=polypoidal choroidal vasculopathy; MAF=minor allele frequency; HWE=*P*-value of Hardy–Weinberg equilibrium test; OR=odds ratio; 95%CI=95% confidence intervals. *The minor allele was calculated based on the all cases and control subjects.

rs42524 was modestly significantly associated with nAMD [p(allelic)=0.0425, minor allele G: 8.04% in nAMD versus 9.47% in the control], but not with PCV [p (allelic)=0.4164, minor allele G: 10.90% in PCV versus 9.47% in the control]. The odds ratio for the G allele of rs42524 was 0.53 (95% CI, 0.28–0.99) for nAMD and 1.21 (95% CI, 0.76–1.92) for PCV (Table 2). The p**value for this association with nAMD was significant under an additive model, but not under a dominant or recessive model. None of the models showed any statistically significant association with PCV (Table 2).

**Table 2:** Association test for the rs42524 genotype in nAMD, PCV and control subjects

**Group**	**Genotype**			**Genotype**	**Genotype distribution (%)**			**p-value**
	Model	OR(95%CI)	P-value		Case	Control	OR(95%CI)	
AMD	Additive	0.5259 (0.2802-0.9869)	0.0454	CC	121(89.0)	147(81.2)	Reference	
	Dominant	0.5360(0.2788-1.0302)	0.0587	GC	15(11.0)	32(17.7)	0.5695(0.2947-1.1005)	0.1105
	Recessive	0.7905(0.6373-0.9805)	0.2188	GG	0	2(1.1)	–	–
							0.5259(0.2802-0.9869)*	0.045*
PCV	Additive	1.2080 (0.7632-1.911)	0.4201	CC	152 (77.9)	147(81.2)	Reference	
	Dominant	1.2231(0.7391-2.0241)	0.4329	GC	40(20.5)	32(17.7)	1.2089(0.7207-2.0277)	0.5126
	Recessive	1.1134(0.8446-1.4679)	0.7139	GG	3(1.5)	2(1.1)	1.4507(0.2389-8.8071)	1
							1.2078(0.7632-1.9115)*	1.2078*

nAMD=neovascular age-related macular degeneration; OR=odds ratio; 95% CI=95% confidence interval; PCV=polypoidal choroidal vasculopathy. * Trend test.

The size of the cohort provides >80% power to detect significant associations (α=0.017) with an effect size index of 0.2 (corresponding to a weak-to-moderate gene effect). The degrees of freedom were 1 for allelic frequencies and 2 for genotype frequencies. The statistical power to detect changes in allelic frequencies for the nAMD and PCV groups versus the controls was 87.98% and 93.21%, respectively, and the power to detect changes in genotype frequencies was 80.66% and 88.01%, respectively.

## Discussion

Both nAMD and PCV are leading causes of blindness and visual impairment in the elderly population. Recently, many studies have found that the two possess different genetic backgrounds, clinical characteristics, and prognoses. These results indicate a strong possibility that PCV and nAMD have different pathogenic mechanisms [32-34].

In recent analyses of human PCV and AMD specimens, several investigations have suggested a possible role for vessel destruction as a pathogenic mechanism in PCV and AMD. Other studies have sought to identify the related SNPs and finally identified a relationship between elastin (*ELN*) and susceptibility to the two diseases [13,19,35].

Collagen is known to decrease the strength of the vascular wall, thus leading to aneurysm formation. Many collagens play important roles in cell adhesion, the maintenance of tissue architecture, and normal tissue function. Among them, *COL1A2* plays an essential role in the expression of collagen type I in vivo, which is important in development and adult tissue repair [36,37]. The *COL1A2* gene is located on chromosome 7q22.1 and has been identified as a susceptibility gene in many collagen-related problems [38,39]. This gene has also been shown to be involved in vascular development, stabilization, maturation, and remodeling [40,41]. Furthermore, many vascular abnormalities, including stroke, myocardial infarction, and IA, have been found to be due to defects in *COL1A2* [28,39,42]. Given the results of these previous studies, we aimed to discover whether *COL1A2* plays a role in the pathogenesis of PCV or neovascular AMD. To our knowledge, this issue has not yet been investigated.

We previously found that PCV was most likely associated with IA and have documented a variant (rs10757278) in 9p21 shared between the two [14]. An investigation in Japanese patients with IA screened the *COL1A2* gene extensively for suspected SNPs and found a particularly strong association between the polymorphism rs42524 and IA under a dominant model [28]. Zhu et al. further confirmed the association between *COL1A2* and IA in a Han Chinese population [43]. Thus, in this study, we aimed to discover whether this SNP plays a role in susceptibility to PCV or nAMD.

The results of our case-control study demonstrated that rs42524 in *COL1A2* is significantly associated with nAMD, which is consistent with recent studies [44,45]. To date, only one group has examined the association between advanced AMD and variants near *COL10A1* (rs1999930) in Caucasian individuals [44]. However, a recent in vitro study found that a reduction in *COL1A2* expression suppressed neovessel growth and curtailed CNV fibrosis [45]. Genome-wide association studies with large cohorts have further strengthened the association between advanced AMD and variants near *COL10A1* (rs1999930) in Caucasian individuals, finding that the development of advanced AMD might be caused in part by extracellular collagen matrix pathways [44].

We found that rs42524, however, is not associated with PCV. This differential susceptibility of PCV and nAMD agrees with previous studies that found little-to-no overlap between PCV and nAMD susceptibility genes [11,12]. Our previous study also showed a lack of association between PCV and *SERPING1* polymorphisms [12]. The same polymorphisms have been shown to have a protective effect for nAMD [46]. Additionally, a common variant (rs10757278) on chromosome 9p21 was reported to be associated with PCV, but not with nAMD, in a Chinese population [14]. Taken together, these findings may indicate that although PCV and nAMD share similar clinical manifestations, the two may be controlled by different collagen genes.

The main limitations of our study included the relatively small sample size and the fact that not all of the collagen genes were surveyed. These results need to be confirmed in a larger cohort and with comprehensive investigations of all of the collagen genes.

In conclusion, we investigated the association of *COL1A2* (rs42524) polymorphisms in PCV and nAMD. We found that rs42524 is significantly associated with nAMD, but not with PCV. This finding may imply that *COL1A2* gene polymorphisms play an important role in the development of nAMD. Finally, we discovered that the G allele of rs42524, rather than the C allele, confers nAMD risk.
